# The Ameliorating Effects of Bushen Huatan Granules and Kunling Wan on Polycystic Ovary Syndrome Induced by Dehydroepiandrosterone in Rats

**DOI:** 10.3389/fphys.2021.525145

**Published:** 2021-03-08

**Authors:** Yang Xu, Chun-Shui Pan, Quan Li, Hao-Lin Zhang, Li Yan, Gulinigaer Anwaier, Xiao-Yi Wang, Lu-Lu Yan, Jing-Yu Fan, Dong Li, Jing-Yan Han

**Affiliations:** ^1^Department of Traditional Chinese Medicine, Peking University Third Hospital, Beijing, China; ^2^Department of Integration of Chinese and Western Medicine, Peking University Health Science Center, Beijing, China; ^3^Tasly Microcirculation Research Center, Peking University Health Science Center, Beijing, China; ^4^Key Laboratory of Stasis and Phlegm, State Administration of Traditional Chinese Medicine, Beijing, China; ^5^State Key Laboratory of Core Technology in Innovative Chinese Medicine, Tianjin, China; ^6^Department of Integration of Chinese and Western Medicine, School of Basic Medical Sciences, Peking University, Beijing, China; ^7^Academy of Integration of Chinese and Western Medicine, Peking University Health Science Center, Beijing, China

**Keywords:** granulosa cells, apoptosis, mitochondria, endoplasmic reticulum stress, traditional Chinese medicine

## Abstract

**Aim:**

To investigate the effects of Bushen Huatan Granules (BHG) and Kunling Wan (KW), the two Chinese medicines, on the regulation of polycystic ovary syndrome (PCOS) and their underlying mechanisms.

**Materials and Methods:**

PCOS rat model was established by subcutaneous injection of dehydroepiandrosterone (DHEA) (6 mg/100 g/day) for 20 days, followed by treatment with BHG (0.75, 1.49, and 2.99 g/kg) or KW (0.46, 0.91, and 1.82 g/kg) by gavage for 4 weeks. Estrous cycle was detected by vaginal smears. Follicles development was assessed by histology. Levels of testosterone and insulin in serum were tested by ELISA. Apoptosis of Granulosa cells (GCs) was evaluated by terminal deoxynucleotidyl transferase-mediated dUTP-biotin nick end-labeling staining. Pathways associated with apoptosis were detected with western blot. Pregnancy outcome was also assessed. GCs were pre-treated with 10^–5^ M testosterone *in vitro* for 24 h, then incubated with serum from rats receiving BHG (1.49 g/kg) or KW (1.82 g/kg). The parameters concerning apoptosis, mitochondrial function and endoplasmic reticulum stress were assessed.

**Results:**

Post-treatment with either BHG or KW ameliorated DHEA-induced irregular estrous cycles, follicles development abnormalities, increase of testosterone and insulin in serum, and the apoptosis of GCs. Post-treatment with BHG decreased the expression of cleaved caspase-9/caspase 9, release of cytochrome C from mitochondria, and mitochondria reactive oxygen species production, increased activities of complex I, II, IV of ovarian tissue. Post-treatment with KW decreased the levels of caspase-12, GRP78, C/EBP homologous protein, phosphorylation of IRE-I, x-box-binding protein 1s, as well as phosphorylation of proline-rich receptor-like protein kinase, phosphorylation of eukaryotic translation initiation factor 2α and ATF4 of ovarian tissue and GCs. Both BHG and KW ameliorated pregnancy outcome.

**Conclusion:**

This study demonstrated BHG or KW as a potential strategy for treatment of PCOS induced by DHEA, and suggested that the beneficial role of the two medicines were mediated by different pathway with the effect of BHG being correlated with the regulation of mitochondria, while the effect of KW being attributable to protection of endoplasmic reticulum stress.

## Introduction

Polycystic ovary syndrome (PCOS) is one of the most common endocrine and metabolic disorders characterized by hyperandrogenism, chronic anovulation and polycystic ovaries, which affects 5–20% of women at reproductive age worldwide (Eser et al., 2017; Yao et al., 2017; Zhong et al., 2018). PCOS accounts for about 70% of anovulatory infertility cases, affecting about 5.6% of women in China (Wu et al., 2015; Li et al., 2019). However, the pathogenesis and etiology of PCOS remain unclear (Wei et al., 2017).

Granulosa cells (GCs) play a crucial role in the growth and development of oocytes and ovarian follicles by providing a suitable microenvironment for them. Bidirectional communication between an oocyte and GCs via regulatory factors is the foundation of follicular growth (Nakamura et al., 2015; Murase et al., 2018). Therefore, dysfunction of GCs has been regarded as one of the key factors that affect the follicles development relevant to PCOS (Matsuda et al., 2012). Apoptosis of GCs in the PCOS patients and PCOS-like animals is noticed in previous studies (Wu et al., 2017; Zheng et al., 2017; Fu et al., 2018; Lima et al., 2018). A feature of PCOS is excessive androgen secretion (Chen et al., 2009), while excessive androgen was reported to promote the development of PCOS by inducing apoptosis and death of GCs (Lim et al., 2017a,b). Previous studies have shown that excessive androgen induced follicular development abnormalities, which promotes preantral follicle growth but suppresses later stages of follicular development (Jonard and Dewailly, 2004; Lee et al., 2018; Azhary et al., 2019). In spite of these findings, the signaling pathways that mediate androgen-induced apoptosis in GCs in PCOS have not been fully identified, which impedes the development of treatment of PCOS (Su et al., 2017; Elkady, 2019).

The standard treatment for PCOS in clinic are selective estrogen receptor modulators clomiphene (Beck et al., 2005), aromatase inhibitors (Casper and Mitwally, 2006) and gonadotropin (Hughes et al., 2000). However, these hormone regulators also have side effects affecting metabolic function and increasing the risk of multiple pregnancies and ovarian hyperstimulation syndrome (Tummon et al., 2005). In addition, there are still some patients who have poor response to ovulation induction therapy and some are still unable to be pregnant after ovulation (Palomba et al., 2006).

Traditional Chinese medicine is widely used in the treatment of PCOS in China. Bushen Huatan Granules (BHG) is an empirical prescription for PCOS, consisting of 10 compositions (Supplementary Material 1), which improves PCOS symptoms and pregnancy outcome in clinic. Kunling Wan (KW) is a standardized Chinese patent medicine for treatment of gynecopathy including PCOS, which consists of 31 compositions (Supplementary Material 2). Our previous study showed that KW has a great efficacy on infertility (Lv et al., 2019). Despite effectiveness of the two medicines in practice, the underlying mechanisms are not explored as yet.

The present study aimed to investigate the effect of BHG and KW, respectively, on the regulation of PCOS and pregnancy outcome induced by dehydroepiandrosterone (DHEA) in rat and their protective effect on the apoptosis of GCs and signaling pathways involved.

## Materials and Methods

### Animals

Sprague Dawley (SD) rats of female, 3-week-old, 50 ± 5 g and male, 10-week-old, 350 ± 20 g were obtained from the Animals Center of Peking University Health Science Center with the certificate number SYXK 2016-0041. The rats were kept in an environment with ambient temperature of 24 ± 1°C and relative humidity of 50 ± 1% with a 12−h light/dark cycle. All animals were fed with standard rat chow and had free access to water and food. The study was carried out in line with the United Kingdom Animal Scientific Procedures Act 1986 and the EU Directive 2010/63/EU for animal experiments. All animals were handled based on the guidelines of the Peking University Animal Research Committee. The Committee on the Ethics of Animal Experiments of Peking University Health Science Center approved the experimental protocol (LA2016314).

### Reagents

Bushen Huatan Granules was supplied by Guangdong Yi Fang Pharmaceutical Co., Ltd. (Guangzhou, GD, China), which consists of 10 compositions (Supplementary Materials 1). KW (approval number: Z21021219, batch number 20180843) was obtained from Tasly Pharmaceutical Co., Ltd. (Fuxing, LN, China), which consists of 31 compositions (Supplementary Materials 2). DHEA was from Cayman Chemical (Ann Arbor, MI, United States). Testosterone was purchased from Wako Pure Chemical Industries (Osaka, Japan). GCs were from Fengmao Technology Co. (Beijing, China). Cell counting kit-8 (CCK-8) was purchased from Beyotime Biotechnology (Shanghai, China). Antibodies against cleaved caspase-9, caspase-8, caspase-12, glucose-regulated protein 78 (GRP78), ATF6, inositol-requiring enzyme 1 (IRE1) and phospho-IRE1 were purchased from Abcam (Cambridge, United Kingdom). Antibodies against Bcl-2 associated X protein (Bax), Bcl-2: B-cell lymphoma-2 (Bcl-2), cleaved caspase-3, caspase-3, caspase-9, cytochrome C, ATF4, x-box-binding protein 1s (XBP1s), proline-rich receptor-like protein kinase (PERK), phospho-PERK, eukaryotic translation initiation factor 2α (eIF2α), phospho-eIF2α, and C/EBP homologous protein (CHOP) were purchased from Cell Signaling Technology (Beverly, MA, United States). Antibody against cytochrome C oxidase subunit 4 isoform 1 (COX4i1) was obtained from Invitrogen Corporation (Camarillo, CA, United States).

### Experiment Protocols

The PCOS model was established as previously reported (Roy et al., 1962). For this, female SD rats were subcutaneously injected daily with DHEA (6 mg/100 g body weight, dissolved in 0.1 mL sesame oil) for 20 consecutive days in the DHEA group. Animals in Control received sesame oil alone the same way. Starting from the 11th day of modeling, estrus cycle was determined every day by vaginal smear. After modeling, blood samples were taken from retroorbital venous plexuses. The animals in DHEA that exhibited disordered estrous cycles and higher level of testosterone than that in Control were either scarified as the group DHEA (3 weeks), or randomly divided into seven groups: DHEA (7 weeks) group, DHEA + BHG (0.75 g/kg), DHEA + BHG (1.49 g/kg), DHEA + BHK (2.99 g/kg), DHEA + KW (0.46 g/kg), DHEA + KW (0.91 g/kg), and DHEA + KW (1.82 g/kg). Rats in the DHEA + BHG groups and DHEA + KW groups were successively administrated daily, respectively, with BHG and KW at the dose indicated by gavage for 28 days. Both KW and BHG were dissolved in normal saline (NS). The doses of the drugs used in the present study were converted from their clinical dosage with low, medium and high dose being half, equivalent and twice of the clinical human dose, respectively, which showed no toxicity for the animals (Supplementary Material 4). Rats in the Control and DHEA (7 weeks) groups were given with NS (10 mL/kg) alone the same way. The number of rats used to determine each parameter in each group is detailed in Table 1.

**TABLE 1:** The number of animals used for assessment of various parameters in each group.

**Control**	**DHEA (3 weeks)**	**DHEA (7 weeks)**	**DHEA + BHG (0.75 g/kg)**	**DHEA + BHG (1.49 g/kg)**	**DHEA + BHG (2.99 g/kg)**	**DHEA + KW (0.46 g/kg)**	**DHEA + KW (0.91 g/kg)**		**Total**
Observation of estrous cycle15	–	15	15	15	15	15	15	15	120
HE staining(3)	3	(3)	(3)	(3)	(3)	(3)	(3)	(3)	3, (24)
Testosterone and insulin in serum(6)	6	(6)	(6)	(6)	(6)	(6)	(6)	(6)	6, (48)
TUNEL staining(3)	(3)	(3)	(3)	(3)	(3)	(3)	(3)	(3)	(27)
MitoSOX(3)	(3)	(3)	(3)	(3)	(3)	(3)	(3)	(3)	(27)
Mitochondrial respiratory chain complexes activities8	8	8	8	8	8	8	8	8	72
Western blot(8)	(8)	(8)	(8)	(8)	(8)	(8)	(8)	(8)	(72)
Pregnancy outcome20	–	18	10	10	9	8	10	10	95
Total43	17	41	33	33	32	31	33	33	296

*Numbers in brackets represent the number of the animals used in the respective experiment, and these animals were among those denoted above by the numbers free of brackets.*

To investigate the ameliorating effects of BHG and KW on infertility of PCOS, some female rats were caged after 28-days-administration of drugs with males at a ratio of 1:1 overnight and checked for vaginal plugs the next morning. Successful mating was confirmed by the presence of vaginal plugs. The pregnant rats were cared until natural delivery. Numbers of mated and delivered rats as well as pups were counted.

### Cell Culture and Treatment

Granulosa cells were cultured in DMEM (Invitrogen, Grand Island, NY, United States) containing 10% fetal bovine serum, at 37°C in a humidified incubator with 95% air-5% CO_2_. GCs were pre-treated with 10^–5^ M testosterone for 24 h, then incubated for further 6 h in the presence of the serum from rats receiving BHG (1.49 g/kg) or KW (1.82 g/kg) and the parameters of interest were assessed.

### Cell Viability Assay

Cell counting kit-8 assay was performed to detect the viabilities of GCs. For this, CCK-8 solution (10 μl) was added to each well, and the plates were incubated for 1 h at 37°C in moist box. The absorbance of each well at 450 nm was measured using Micro-Plate Reader (Bio-Rad, Hercules, CA, United States). Cell viability was quantified using average absorbance × 100%.

### Ovary Index and Histological Evaluation of Ovarian

Ovaries were harvested from rats under anesthesia by intramuscular injection with 20% urethane. The ovary index (ovary wet weight/body weight) was determined and expressed as mg/g. Ovaries were fixed in 4% paraformaldehyde for 48 h and processed for paraffin sections as routine. Ten successive sections of 5 μm with an interval of 50 μm between each were collected from each ovary and stained with hematoxylin and eosin (HE), on which the follicles were classified and counted under a light microscope (BX512DP70; Olympus, Tokyo, Japan). The follicles at different development stages were defined as follows: primordial follicle, an oocyte surrounded by a layer of flat GCs; primary follicle, an oocyte surrounded by a layer of cubic GCs; secondary follicle, an oocyte surrounded by more than two layers of granular cells without follicular cavity; antral follicle, an oocyte with one or multiple follicular cavities and multiple layers of granular cells as well as cumulus GC layer. Nuclei was visible in oocyte; cystic follicle, a big cavity with a thin layer of granular cells and obvious theca cells without oocyte; corpus luteum, gland-like structure enriched in blood vessels.

### Determination of Body Weight, Fasting Blood Glucose, HOMA Index, Testosterone and Insulin

The blood of rat was collected at the end of the study from the abdominal aorta or orbital vein. Serum samples were obtained by centrifugation at 2054 *g* for 20 min at 4°C and stored at −80°C before analysis. The levels of testosterone and insulin in serum were tested by using ELISA kits (Abcam, Cambridge, United Kingdom) in accordance with the manufacturer’s instructions. Fasting Blood Glucose and HOMA Index were tested as routine.

### Terminal Deoxynucleotidyl Transferase-Mediated dUTP-Biotin Nick End-Labeling Staining

Terminal deoxynucleotidyl transferase-mediated dUTP-biotin nick end-labeling (TUNEL) staining was performed on paraffin sections of rat ovary with a TUNEL kit (Roche, Basel, Switzerland) in accordance with the manufacturers protocol. Nuclei were stained with Hoechst 33342 (Invitrogen, Camarillo, CA, United States). TUNEL positive cells were counted on randomly selected five fields in each section, under a laser scanning confocal microscope (TCS SP8; Leica, Mannheim, Germany), one sections from each animal. The average was calculated and presented as cell number per field.

### Quantification of ROS Production in Mitochondria of Ovarian Tissue

To assess reactive oxygen species (ROS) production, ovarian sections were incubated for 15 min at 37°C in the dark with MitoSOX-Red (Invitrogen, Camarillo, CA, United States), a fluorochrome specific for superoxide produced in mitochondria. Nuclei were stained with Hoechst 33342. Images were grasped by using a confocal microscopy (TCS SP8; Leica, Mannheim, Germany). For quantitative analysis, three sections were chosen in each group and five fields were randomly selected per section. Analysis was conducted by using Image-Pro Plus 6.0 software (Media Cybernetics, Bethesda, MD, United States).

### Determination of Activities of Mitochondrial Respiratory Chain Complexes

The ovarian tissue was harvested and immediately frozen in liquid nitrogen and stored at −80°C till use. GCs were washed with PBS for three times and stored at −80°C till use. In accordance with the manufacturer’s protocol, mitochondrial respiratory chain Complex I, Complex II, and Complex IV activities in tissues and GCs were measured using ELISA kits (Abcam, Cambridge, United Kingdom). Briefly, samples were homogenized, pelleted and adjusted to 5.5 mg/ml, subjected to detergent extraction and centrifugation for 20 min at 16,000 *g* (for Complex I), 12 000 g (for Complex II), and 24,127*g* (for Complex IV), followed by loading supernatant on plate and incubation for 3 h. Following washing for three times, optical density was measured after addition of assay solution.

### Western Blot Assay

Rats under anesthesia were sacrificed at the end of the experiment, the ovarian tissue was harvested and immediately frozen in liquid nitrogen and stored at −80°C till use. The whole protein of the tissues and GCs were homogenized in RIPA lysis buffer with protease inhibitor (Applygen Technologies, Beijing, China). The isolation of cytoplasmic and mitochondrial proteins in tissues and GCs was completed according to the Mitochondrial Isolation kit instructions (Abcam, Cambridge, United Kingdom). Protein samples were separated by sodium dodecyl sulfate−polyacrylamide gel electrophoresis and transferred to polyvinylidene fluoride membrane. Blots were blocked with 5% fat-free milk or bovine serum albumin according to the antibody supplier’s recommendation in Tris−buffered saline Tween, and membranes were incubated over night at 4°C with primary antibodies against Bax (1:1000), Bcl-2 (1:1000), cleaved caspase-3 (1:1000), caspase-3 (1:2000), cleaved caspase-9 (1:1000), caspase-9 (1:1000), caspase-8 (1:6000), caspase-12 (1:1000), cytochrome C (1:1000), COX4i1 (1:1000), GRP78 (1:1000), CHOP (1:1000), ATF6 (1:1000), IRE1 (1:1000) and phospho-IRE1 (1:1000), XBP1s (1:1000), PERK (1:1000), phospho-PERK (1:1000), eIF2α(1:1000), phospho-eIF2α (1:1000), and ATF4 (1:1000) and GAPDH (1:5000). After washed with TBST for three times, the membranes were incubated with secondary antibody (1:5000) for 1 h at room temperature and washed again by TBST for three times. GAPDH was used as a loading control for cytoplasmic proteins, and COX4i1 was used as a loading control for mitochondria proteins. Enhanced Chemiluminescence Detection Kit (Applygen Technologies, Beijing, China) was used for revealing the bands. Band intensities were quantified by densitometry and presented as mean area density using Quantity One image analyzer software (Bio-Rad; Richmond, CA, United States).

### Data Analysis

Statistical significances between groups were determined using one-way ANOVA with Bonferroni test. Data were expressed as mean ± SEM with *P* < 0.05 considered statistically significant.

## Results

### BHG and KW, Respectively, Ameliorates Rat Estrous Cycle Dysfunction Induced by DHEA

**FIGURE 1:**
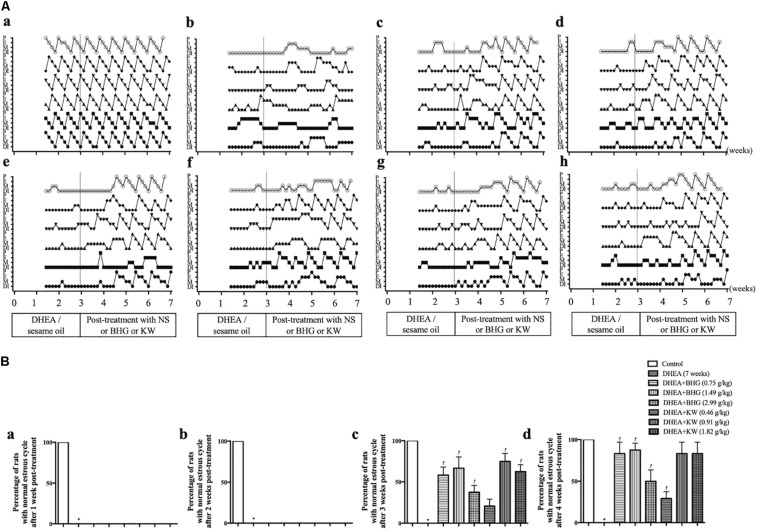
Effect of post-treatment with BHG or KW on rat estrous cycle. **(A)** Representative depictions illustrating the changes of estrous cycle in Control **(a)**, DHEA (7 weeks) **(b)**, DHEA + BHG (0.75 g/kg) **(c)**, DHEA + BHG (1.49 g/kg) **(d)**, DHEA + BHG (2.99 g/kg) **(e)**, DHEA + KW (0.46 g/kg) **(f)**, DHEA + KW (0.91 g/kg) **(g)**, and DHEA + KW (1.82 g/kg) **(h)** group, respectively. P, preoestrus; E, estrus; M, metaoestrus; DI, diestrus. **(B)** Statistical result of the percentage of rats with normal estrous cycle after treatment with BHG or KW for 1, 2, 3, and 4 weeks. Results are presented as mean ± SEM. *P < 0.05 vs. Control group; ^†^*P* < 0.05 vs. DHEA (7 weeks) group, *n* = 15.

The estrous cycles of rats were examined from the 11th day of modeling to the day the rats were killed. Representative records of estrous cycles of different groups are illustrated in Figure 1A. All rats in the Control group (Figure 1Aa) had normal estrous cycles. DHEA induced irregular estrous cycles, which persisted till the end of the study, if not intervened (Figure 1Ab). Post-treatment with BHG (0.75, 1.49, and 2.99 g/kg) or KW (0.46, 0.91, and 1.82 g/kg) all recovered the irregular estrous cycles form the third week of the administration (Figure 1Ac–h). Figure 1B and Table 2 are the statistical results of the percentage of rats with normal estrous cycles after treatment with BHG or KW in different groups, which further confirmed the results of the estrous cycles records.

**TABLE 2:** Number and percentage of the follicles in different development stages and corpus luteum.

	**Control**		**DHEA (3 weeks)**		**DHEA (7 weeks)**		**DHEA + BHG (0.75 g/kg)**		**DHEA + BHG (1.49 g/kg)**		**DHEA + BHG (2.99 g/kg)**		**DHEA + KW (0.46 g/kg)**		**DHEA + KW (0.91 g/kg)**		**DHEA + KW (1.82 g/kg)**	
	**Number**	**Per-centage**	**Number**	**Per-centage**	**Number**	**Per-centage**	**Number**	**Per-centage**	**Number**	**Per-centage**	**Number**	**Per-centage**	**Number**	**Per-centage**	**Number**	**Per-centage**	**Number**	**Per-centage**
Primordial follicle	183.67 ± 6.27	48.23 ± 0.8	142.67 ± 13.13	39.33 ± 1.45*	144 ± 0.8	42 ± 1.73*	151.33 ± 9.86	43.67 ± 0.88	162.67 ± 10.78	43.17 ± 0.60	140.67 ± 8.63	39.67 ± 0.67	147.67 ± 5.84	38.67 ± 0.88	148.67 ± 9.29	40 ± 0.58	156.33 ± 9.74	42 ± 0.58
Primary follicle	118 ± 3.28	31.03 ± 0.32	134.33 ± 4.8	37.2 ± 0.71*	119.33 ± 5.76	34.67 ± 0.33*	107.67 ± 7.28	31.13 ± 1.07^#†^	109.33 ± 4.4	29 ± 0.58^#†^	113.67 ± 7.98	32 ± 0.58^#^	121.33 ± 7.11	31.67 ± 0.48^#^	118 ± 6.6	31.73 ± 0.48^#^	113 ± 7.06	30.4 ± 0.70^#†^
Secondary follicle	18.67 ± 1.21	4.93 ± 0.23	22.33 ± 1.29	6.23 ± 0.18	21 ± 2.08	6.13 ± 0.37	18 ± 0.72	5.17 ± 0.22	22.67 ± 2.01	6.03 ± 0.26	17.67 ± 2.06	5.03 ± 0.26	20 ± 0.39	5.19 ± 0.24	18.67 ± 0.75	5.07 ± 0.18	21.67 ± 0.99	5.79 ± 0.27
Antral follicle	48.67 ± 2.43	12.8 ± 0.42	31.67 ± 1.82	8.77 ± 0.12*	30 ± 1.78	8.73 ± 0.15*	42.67 ± 1.38	12.33 ± 0.44^#†^	49.67 ± 5.33	13.17 ± 1.17^#†^	43.67 ± 4.90	12.38 ± 0.61^#†^	45 ± 0.43	11.67 ± 0.35^#†^	45.67 ± 3.2	12.27 ± 0.15^#†^	49.33 ± 3.08	13.27 ± 0.37^#†^
Cystic follicle	5.67 ± 0.19	1.45 ± 0.03	24.67 ± 1	6.8 ± 0.42*	24.33 ± 1.62	7.07 ± 0.29*	16 ± 1.21	4.6 ± 0.1^#†^	16.33 ± 1.76	4.3 ± 0.25^#†^	18.33 ± 2.09	5.2 ± 0.36^†^	20 ± 1.45	5.2 ± 0.36^†^	17.33 ± 2.1	4.6 ± 0.23^#†^	18 ± 2.2	4.9 ± 0.38^#†^
Total count	380.67 ± 6.74		361.67 ± 19.77		344 ± 15.72		346.67 ± 18.59		376.67 ± 20		355 ± 22.14		382.67 ± 14.75		372.33 ± 26.6		371.67 ± 20.92	
Corpus luteum	68.67 ± 1.76		26.67 ± 0.88*		23 ± 1.15*		48 ± 1.73^#†^		49 ± 2.4^#†^		39.33 ± 2.4^#†^		48.67 ± 1.67^#†^		47.67 ± 4.26^#†^		40.33 ± 1.86^#†^	

Statistical result of the number and percentage of the follicles in different development stages and corpus luteum in various groups. Results are presented as mean ± SE. *P < 0.05 vs. Control group; ^#^*P* < 0.05 vs. DHEA (3 weeks) group; ^†^*P* < 0.05 vs. DHEA (7 weeks) group, *n* = 15.

### BHG and KW, Respectively, Ameliorates DHEA-Induced Follicles Development Abnormalities and Reduced Cystic Follicles Formation

**FIGURE 2:**
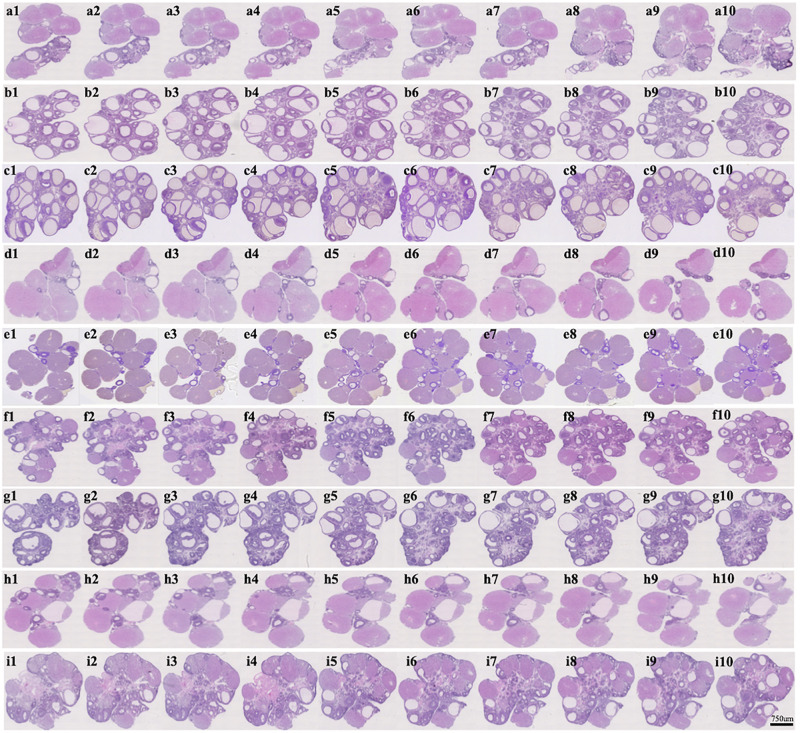
Effect of post-treatment with BHG or KW on histology of the ovarian tissue. Representative images of 10 continuous HE staining sections of rat ovarian tissue in Control **(a1–a10)**, DHEA (3 weeks) **(b1–b10)**, DHEA (7 weeks) **(c1–c10)**, DHEA + BHG (0.75 g/kg) **(d1–d10)**, DHEA + BHG (1.49 g/kg) **(e1–e10)**, DHEA + BHG (2.99 g/kg) **(f1–f10)**, DHEA + KW (0.46 g/kg) **(g1–g10)**, DHEA + KW (0.91 g/kg) **(h1–h10)**, and DHEA + KW (1.82 g/kg) **(i1–i10)** group, respectively.

**FIGURE 3:**
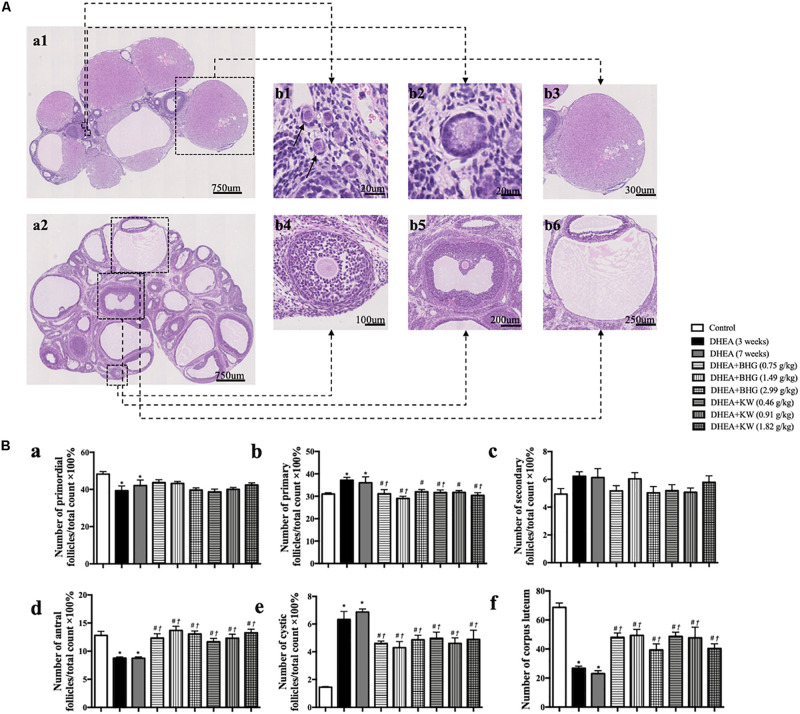
Effect of post-treatment with BHG or KW on follicle development of the ovary. **(A)** Representative HE stained images of rat ovarian tissue **(a1, a2)**. Shown on right side are the representative images of primordial follicle **(b1)**, primary follicle **(b2)**, secondary follicle **(b4)**, antral follicle **(b5)**, cystic follicle **(b6)**, and corpus luteum **(b3)**, respectively. **(B)** Statistical result of the percentage of the follicles in different development stages and the number of corpus luteum in various groups. Results are presented as mean ± SEM. *P < 0.05 vs. Control group; ^#^*P* < 0.05 vs. DHEA (3 weeks) group; ^†^*P* < 0.05 vs. DHEA (7 weeks) group, *n* = 15.

The representative histological images from different groups are presented in Figure 2, which shows that the numbers of cystic follicles were significantly increased in DHEA (3 weeks) group (Figure 2b) and DHEA (7 weeks) group (Figure 2c) compared with the Control group (Figure 2a). Obviously, post-treatment with BHG (0.75, 1.49, and 2.99 g/kg) or KW (0.46, 0.91, and 1.82 g/kg) all reduced the number of cystic follicles to varying degree (Figure 2d–i). Figure 3A is the representative images of follicles at different developmental stages and corpus luteum. Figure 3B and Table 2 are the percentage and number of the different follicles and corpus luteum in different groups, demonstrating that the percentage of primary follicles in DHEA (3 weeks) group and DHEA (7 weeks) group was increased, which was ameliorated by BHG and KW. In DHEA (3 weeks) group and DHEA (7 weeks) group, the percentage of antral follicles and the number of corpus luteum both significantly decreased, while the percentage of cystic follicles significantly increased. These results suggest that DHEA promotes early follicular growth and suppresses late follicular development. Post-treatment with BHG (0.75, 1.49, and 2.99 g/kg) or KW (0.46, 0.91, and 1.82 g/kg) all relieved abnormalities mentioned above, indicating the ameliorating effect of BHG and KW on follicles development abnormalities and cystic follicles formation.

### BHG and KW, Respectively, Relieves DHEA-Induced Increase in Ovary Index, Testosterone in Serum and Metabolism Disorder

**FIGURE 4:**
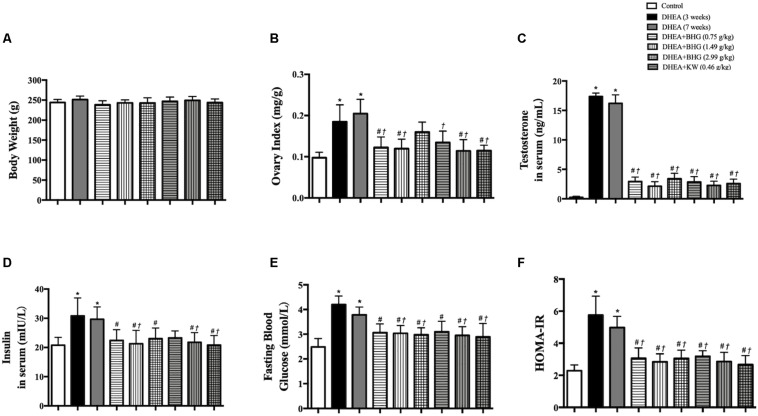
Effect of post-treatment with BHG or KW on ovary index (ovary wet weight/body weight), the level of testosterone and insulin in rat serum and metabolism disorder. **(A)** The body weight in different group. **(B)** Ovary index (mg/g) of various groups. **(C)** Testosterone level in serum in various groups. **(D)** Insulin level in serum in various groups. **(E)** Fasting blood glucose in various groups. **(F)** HOMA index in various groups. Results are presented as mean ± SEM. *P < 0.05 vs. Control group; ^#^*P* < 0.05 vs. DHEA (3 weeks) group; ^†^*P* < 0.05 vs. DHEA (7 weeks) group, *n* = 15 for testosterone level, *n* = 8 for others.

In line with the follicles development abnormalities and increased cystic follicles formation, DHEA, though had no effect on body weight (Figure 4A), also increased the ovary index as compared with Control group, while treatment with BHG (0.75 and 1.49 g/kg) and KW (0.46, 0.91, and 1.82 g/kg) both relieved this alteration (Figure 4B). Testosterone increased immediately after modeling and lasted until the end of the experiment if not interfered. The increased level of testosterone was prevented by treatment with BHG and KW at all doses (Figure 4C). Similarly, the level of insulin in serum (Figure 4D), fasting blood glucose (Figure 4E), and HOMA index (Figure 4F) increased after modeling and lasted for 4 weeks until the rats were scarified. These increases were prevented by treatment with BHG and KW.

### BHG and KW, Respectively, Reduces the Apoptosis of Granulosa Cells Induced by DHEA

**FIGURE 5:**
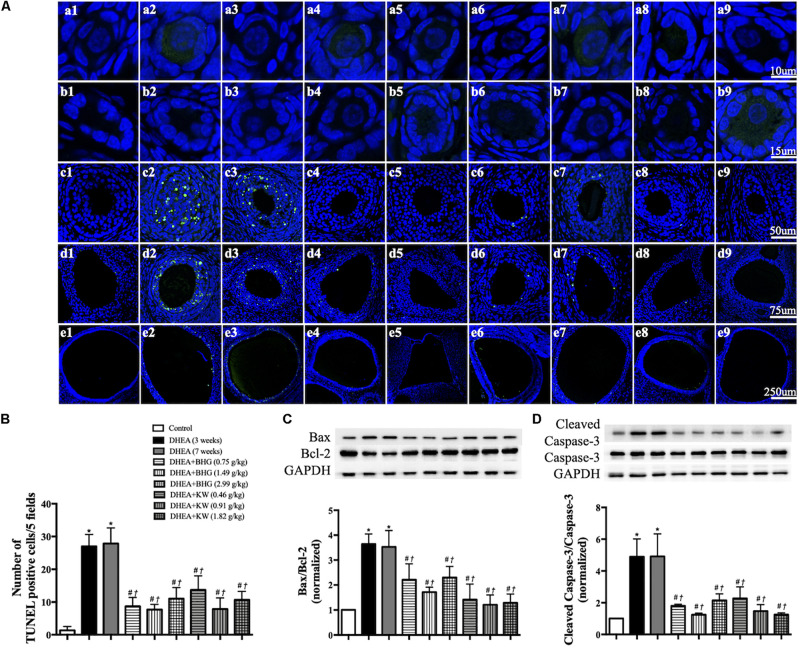
Effect of post-treatment with BHG or KW on apoptosis of granulosa cells in ovarian tissue. **(A)** Representative TUNEL stained images of primordial follicle **(a)**, primary follicle **(b)**, secondary follicle **(c)**, antral follicle **(d)**, and cystic follicle **(e)** in Control **(1)**, DHEA (3 weeks) **(2)**, DHEA (7 weeks) **(3)**, DHEA + BHG (0.75 g/kg) **(4)**, DHEA + BHG (1.49 g/kg) **(5)**, DHEA + BHG (2.99 g/kg) **(6)**, DHEA + KW (0.46 g/kg) **(7)**, DHEA + KW (0.91 g/kg) **(8)**, and DHEA + KW (1.82 g/kg) **(9)**, group respectively. TUNEL positive cells are stained green. **(B)** Statistical result of the number of TUNEL positive cells per five fields in different groups. **(C)** Western blot for the expression of Bax to Bcl-2 in different groups with the quantification of Bax/Bcl-2 showing below. **(D)** Western blot for the expression of cleaved caspase-3 and caspase-3 in different groups with the quantification of cleaved caspase-3/caspase-3 showing below. Results are presented as mean ± SEM. *P < 0.05 vs. Control group; ^#^*P* < 0.05 vs. DHEA (3 weeks) group; ^†^*P* < 0.05 vs. DHEA (7 weeks) group, *n* = 3 for TUNEL staining, *n* = 8 for western blot.

We next performed TUNEL staining on ovarian sections from various groups (Figure 5A). Apoptotic GCs were not noticed in Control group, but observed obviously in secondary follicle (Figure 5A, c2, c3), antral follicle (Figure 5A, d2, d3) and cystic follicle (Figure 5A, e2, e3) in DHEA (3 weeks) group and DHEA (7 weeks) group. The treatment with BHG and KW at all doses prevented the apoptosis in all type of follicles after DHEA (Figure 5A, c4 through 9, d4 through 9 and e4 through 9). These results were confirmed by statistical analysis (Figure 5B). Assessment by western blot provided further support for the results above, with the ratios of Bax to Bcl-2 and cleaved caspase-3 to caspase-3 varying among groups in a similar fashion as the TUNEL positive cells did (Figures 5C,D).

### BHG and KW, Respectively, Decreases the Apoptosis Through Different Pathways

**FIGURE 6:**
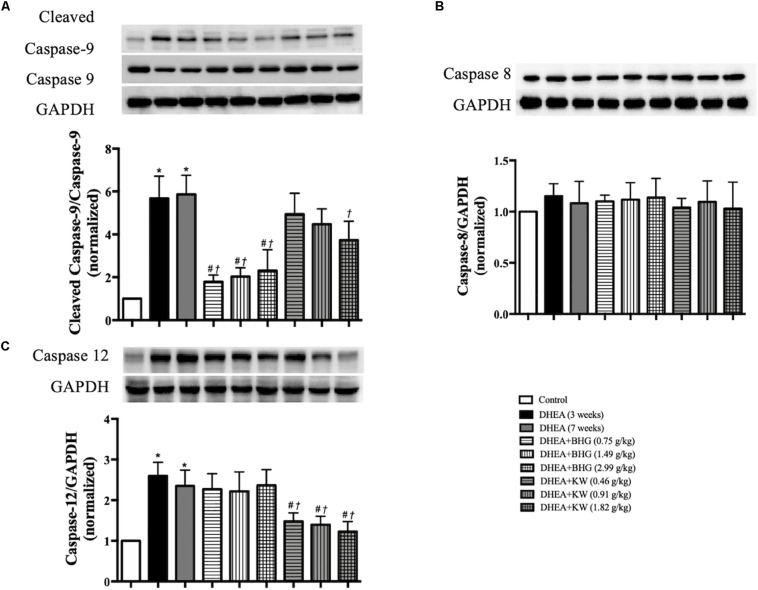
Effect of post-treatment with BHG or KW on the apoptosis pathways. **(A)** Western blot for the expression of cleaved caspase-9 to caspase-9 in different groups with the quantification of cleaved caspase-9/caspase-9 showing below. **(B)** Western blot for the expression of caspase-8 in different groups with the quantification of caspase-8 showing below. **(C)** Western blot for the expression of caspase-12 in different groups with the quantification of caspase-12 showing below. Results are presented as mean ± SEM. *P < 0.05 vs. Control group; ^#^*P* < 0.05 vs. DHEA (3 weeks) group; ^†^*P* < 0.05 vs. DHEA (7 weeks) group, *n* = 8.

To explore the signaling pathways implicated in the role of the two drugs in attenuating the apoptosis of GCs after DHEA, western blots were carried out to assess the expression of cleaved caspase-9, caspase-9, caspase-8, and caspase-12 in ovarian tissue from various groups. As shown in Figures 6A–C, compared with the Control group, expression of the ratio of cleaved caspase-9 to caspase-9 and caspase-12 significantly increased in the DHEA (3 weeks) group and DHEA (7 weeks) group, while the expression of caspase-8 did not vary significantly among groups, indicating that the mitochondrial-dependent and endoplasmic reticulum (ER) stress pathways, but not death receptor-dependent pathway, were involved in the DHEA-induced GCs apoptosis. The increase in the expression of the ratio of cleaved caspase-9 to caspase-9 was significantly reduced by BHG, but not KW, at all the three doses tested. On the other hand, the increase in the expression of caspase-12 was significantly reduced by KW, but not BHG, at all the three doses. The results suggest that BHG and KW protected the DHEA-induced apoptosis, respectively, through different pathway.

### BHG Attenuates the Dysfunctional Mitochondria After DHEA

**FIGURE 7:**
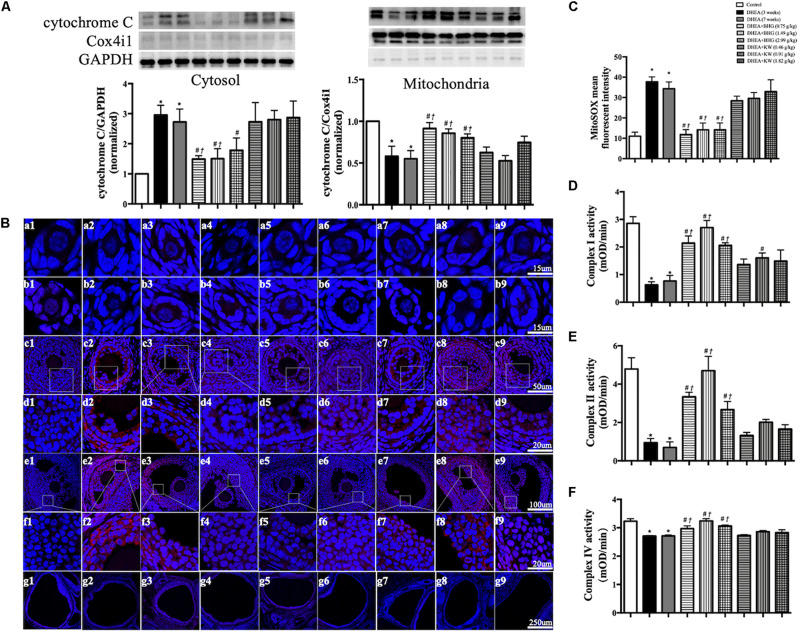
Effect of BHG post-treatment on the dysfunction in mitochondria. **(A)** Representative immunoblot images of cytochrome C in cytosol and mitochondria with the quantification of cytochrome C/GAPDH and cytochrome C/Cox4i1 showing below. GAPDH and Cox4i1 were used as loading control for cytosolic and mitochondrial protein, respectively. **(B)** Representative confocal images of MitoSOX staining (red) for mitochondrial ROS production. Shown are primordial follicle **(a)**, primary follicle **(b)**, secondary follicle **(c)**, enlarged images of secondary follicle **(d)**, antral follicle **(e)**, enlarged images of antral follicle **(f)**, and cystic follicle **(g)** in Control **(1)**, DHEA (3 weeks) **(2)**, DHEA (7 weeks) **(3)**, DHEA + BHG (0.75 g/kg) **(4)**, DHEA + BHG (1.49 g/kg) **(5)**, DHEA + BHG (2.99 g/kg) **(6)**, DHEA + KW (0.46 g/kg) **(7)**, DHEA + KW (0.91 g/kg) **(8)**, and DHEA + KW (1.82 g/kg) **(9)** group, respectively. Nuclei are stained in blue. **(C)** Quantification of MitoSOX mean fluorescent intensity in different group. **(D–F)** Statistical results of complex I, II, IV activities of ovarian tissue from different groups tested by ELISA. Results are presented as mean ± SEM. *P < 0.05 vs. Control group; ^#^*P* < 0.05 vs. DHEA (3 weeks) group; ^†^*P* < 0.05 vs. DHEA (7 weeks) group, *n* = 3 for MitoSOX staining, *n* = 8 for western blot and complex activity analysis.

The signaling pathway involved in the protective effect of BHG on apoptosis suggests occurrence of dysfunction of mitochondria in response to DHEA. To verify this inference, we first tested the cytochrome C release from mitochondria to cytosol by western blot in different groups, since one of the manifestations of dysfunctional mitochondria is open of mitochondrial permeability transition pore (mPTP). As shown in Figure 7A, the expression of cytochrome C in DHEA (3 weeks) group and DHEA (7 weeks) group increased in the cytosol fraction, compared to the mitochondrial fraction, indicating release of cytochrome C from the mitochondria into the cytosol. This change was protected by BHG, but not by KW. Release of mitochondrial cytochrome C into the cytosol is a known feature of the mitochondrial-dependent pathway of apoptosis (Allagnat et al., 2013). Dysfunctional mitochondria may manifest malregulated respiratory chain and oxidative stress alike. We thus tested mitochondrial ROS production in different groups with the MitoSOX probe. The production of ROS in mitochondria increased in DHEA (3 weeks) group and DHEA (7 weeks) group, which was reduced by BHG but not KW (Figures 7B,C). We next detected the activities of mitochondrial complex I (Figure 7D), II (Figure 7E), and IV (Figure 7F), and found that all of the mitochondrial complexes activities detected showed a significant decrease in DHEA (3 weeks) group and DHEA (7 weeks) group, and were ameliorated by post-treatment of BHG at all doses but not KW. These findings suggested that DHEA challenge impaired mitochondria thus initiated mitochondrial-dependent apoptotic signaling pathway, which was ameliorated by BHG post-treatment.

### KW Relieves the ER Stress After DHEA Stimulation

**FIGURE 8:**
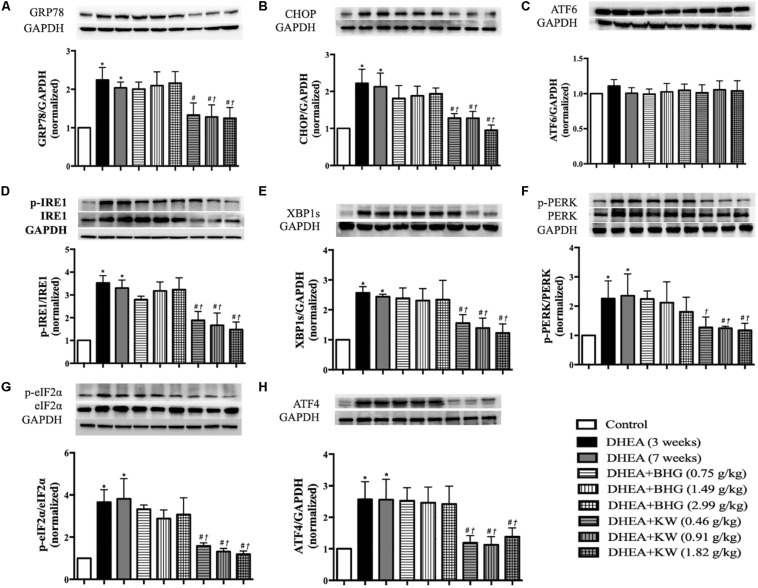
Effect of KW post-treatment on ER stress. **(A–H)** Presented are the representative western blotting images of GRP78, CHOP, ATF6, phosphorylation-inositol-requiring enzyme 1(p-IRE1), IRE1, XBP1s, phosphorylation-proline-rich receptor-like protein kinase (p-PERK), PERK, phosphorylation- eukaryotic translation initiation factor 2α (p-eIF2α), eIF2α, and ATF4 with the quantification showing below. Results are presented as mean ± SEM. *P < 0.05 vs. Control group; ^#^*P* < 0.05 vs. DHEA (3 weeks) group; ^†^*P* < 0.05 vs. DHEA (7 weeks) group, *n* = 8 for western blot.

Increase in the expression of caspase-12 following DHEA stimulation is indicative of ER stress. To further bear out this assumption and examine the role of KW in DHEA-caused ER stress, we tested the expression of GRP78 and CHOP, two marker proteins for the ER stress level. As shown in Figures 8A,B, compared with the Control group, DHEA (3 weeks) group and DHEA (7 weeks) group showed increased expression of GRP78 and CHOP, which was protected by post-treatment of KW at all doses but not BHG. We next examined by western blot which of the three ER stress sensors (ATF6, IRE1, and PERK) mediated the apoptosis of GCs. As shown in Figure 8C, the expression of ATF6 did not differ obviously among groups. In contrast, the ratio of phospho-IRE1 to IRE1 and XBP1s expression were upregulated significantly after modeling, which lasted until the end of the experiment if not interfered. After treatment of KW at all doses, the expression of phospho-IRE1 to IRE1 and XBP1s was significantly decreased (Figures 8D,E). The ratio of phospho-PERK to PERK, phospho-eIF2α to eIF2α, and ATF4 expression varied among groups in a similar manner (Figures 8F–H). The results suggested it is the IRE1-XBP1s and PERK-eIF2α-ATF4, but not ATF6, that were involved in the DHEA-induced ER stress, which were ameliorated by KW post-treatment. Whereas BHG did not show effect on this signaling.

### BHG and KW, Respectively, Improves Pregnancy Outcome of DHEA-Induced PCOS

Polycystic ovary syndrome is one of the most common causes of female infertility, and approximately 74% of PCOS patients are infertile (Li et al., 2017, 2019). We investigated the ameliorating effect of BHG and KW, respectively, on pregnancy outcome of the DHEA-induced PCOS rats. Results showed that the percentage of delivered number of rats obviously decreased in the DHEA (7 weeks) group, while BHG and KW, respectively, improved the delivered rate. Compared with the Control group, the number of pups significantly reduced in the DHEA (7 weeks) group and improved significantly after post-treatment of BHG and KW (Table 3).

**TABLE 3:** Effects of BHG and KW, respectively, on pregnancy outcome in rats.

**Groups**	**Examined**	**Percentage of mated number of rats (%)**	**Percentage of delivered number of rats (%)**	**Number of pups**
Control	20	17 (85.0)	11 (64.7)	10.36 ± 0.64
DHEA (7 weeks)	18	7 (38.9)	2 (28.6)	4.50 ± 0.50*
DHEA + BHG (0.75 g/kg)	10	8 (80.0)	3 (37.5)	9.33 ± 0.88^†^
DHEA + BHG (1.49 g/kg)	10	9 (90.0)	5 (55.6)	9.8 ± 1.07^†^
DHEA + BHG (2.99 g/kg)	9	7 (77.8)	3 (42.9)	8.33 ± 1.20^†^
DHEA + KW (0.46 g/kg)	8	7 (87.5)	4 (57.1)	8.75 ± 1.25^†^
DHEA + KW(0.91 g/kg)	10	8 (80.0)	4 (50.0)	9.5 ± 1.76^†^
DHEA + KW (1.82 g/kg)	10	9 (90.0)	5 (55.6)	10.6 ± 1.08^†^

*Statistics result of pregnancy outcome in different groups. Results are presented as mean ± SEM. ^∗^*P* < 0.05 vs. Control group; ^†^*P* < 0.05 vs. DHEA (7 weeks) group.*

### BHG and KW Ameliorates the GCs Viability and Reduces the Apoptosis of GCs Induced by Testosterone

**FIGURE 9:**
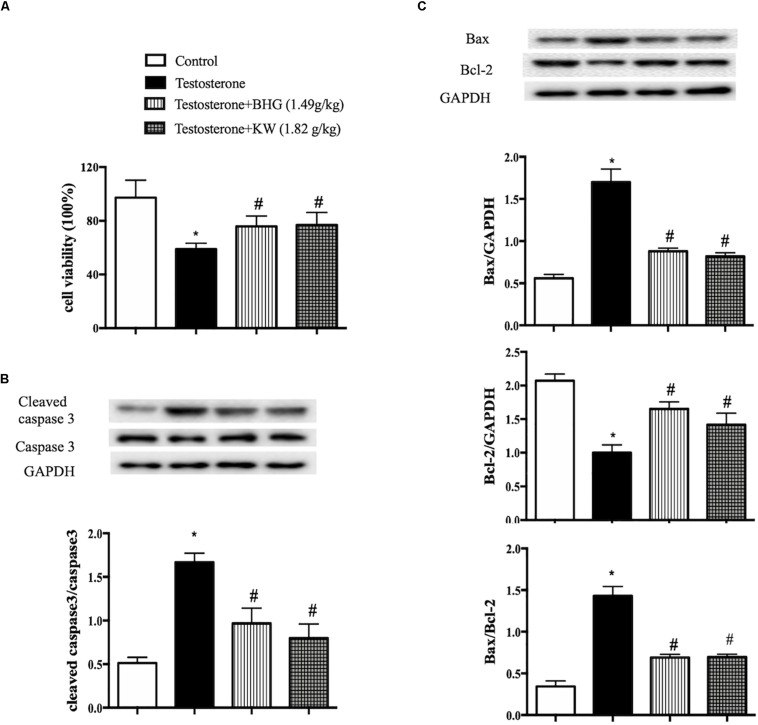
Effect of post-treatment with BHG or KW on the cell viability and apoptosis of GCs induced by testosterone. **(A)** CCK-8 detection for GCs viability in different groups. **(B)** Western blot for the expression of cleaved caspase-3 and caspase-3 in different groups with the quantification of cleaved caspase-3/caspase-3 showing below. **(C)** Western blot for the expression of Bax, Bcl-2 in different groups with the quantification showing below. Results are presented as mean ± SEM. *P < 0.05 vs. Control group; ^#^*P* < 0.05 vs. Testosterone group, *n* = 6 for CCK-8, *n* = 3 for western blot. Testosterone + BHG (1.49 g/kg) denotes the cells treated with serum from animals receiving BHG at 1.49 g/kg, Testosterone + KW (1.82 g/kg) denotes the cells treated with serum from animals receiving KW at 1.82 g/kg.

The findings in PCOS model were verified *in vivo* in GCs. For this purpose, we first detected the effects of BHG and KW on viability and apoptosis of GCs induced by testosterone. As compared with control group, testosterone markedly decreased viability, while both BHG and KW ameliorated GCs viability significantly after testosterone (Figure 9A). Western blot assessment showed that testosterone increased the ratios of cleaved caspase-3/caspase-3 and Bax/Bcl-2, elevated the level of Bax and decreased the level of Bcl-2 in GCs, while both BHG and KW ameliorated these alterations (Figures 9B,C).

### BHG Decreases the Apoptosis Through Attenuating the Dysfunctional Mitochondria of GCs Induced by Testosterone

**FIGURE 10:**
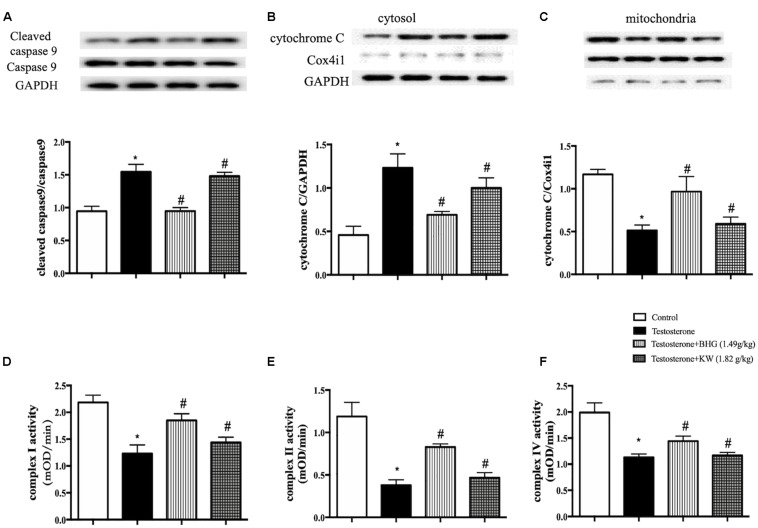
Effect of BHG on the dysfunction in mitochondria of GCs induced by testosterone. **(A)** Western blot for the expression of cleaved caspase-9 and caspase-9 in different groups with the quantification of cleaved caspase-9/caspase-9 showing below. **(B)** Representative western blot images of cytochrome C in cytosol form various groups with quantification showing below. **(C)** Representative western blot images of cytochrome C in mitochondria from various groups with the quantification showing below. GAPDH and Cox4i1 were used as loading control for cytosolic and mitochondrial protein, respectively. **(D–F)** Statistical results of complex I, II, IV activities of GCs from different groups tested by ELISA. Results are presented as mean ± SEM. *P < 0.05 vs. Control group; ^#^*P* < 0.05 vs. Testosterone group, *n* = 3 for western blot and *n* = 6 for complex activity analysis. Testosterone + BHG (1.49 g/kg) denotes the cells treated with serum from animals receiving BHG at 1.49 g/kg, Testosterone + KW (1.82 g/kg) denotes the cells treated with serum from animals receiving KW at 1.82 g/kg.

Western blots were carried out to assess the expression of cleaved caspase-9 and caspase-9 to explore the signaling pathways implicated in the apoptosis of GCs induced by testosterone. The results showed that testosterone induced the increase of cleaved caspase-9/caspase 9 in GCs. The increase ratio of cleaved caspase-9/caspase 9 was significantly reduced by BHG, but not KW (Figure 10A), indicating involvement of ameliorating dysfunctional mitochondria in the effect of BHG on apoptosis after testosterone. We next tested the function of mitochondria in different groups. As shown in Figures 10B,C, compared with the Control, the expression of cytochrome C in testosterone group increased in the cytosol fraction, but decreased in mitochondrial fraction, indicating release of cytochrome C from the mitochondria into the cytosol. BHG ameliorated this alteration, while KW had no effect. We next detected the activities of mitochondrial complex I, II, and IV and found that all of the mitochondrial complexes activities detected showed a significant decrease in testosterone group, which were ameliorated by BHG, but not by KW (Figures 10D–F). These findings suggested that BHG ameliorated the mitochondrial-dependent apoptotic signaling pathway induced by testosterone whereas KW did not show effect on this signaling.

### KW Decreases the Apoptosis Through Relieving the ER Stress of GCs Induced by Testosterone

**FIGURE 11:**
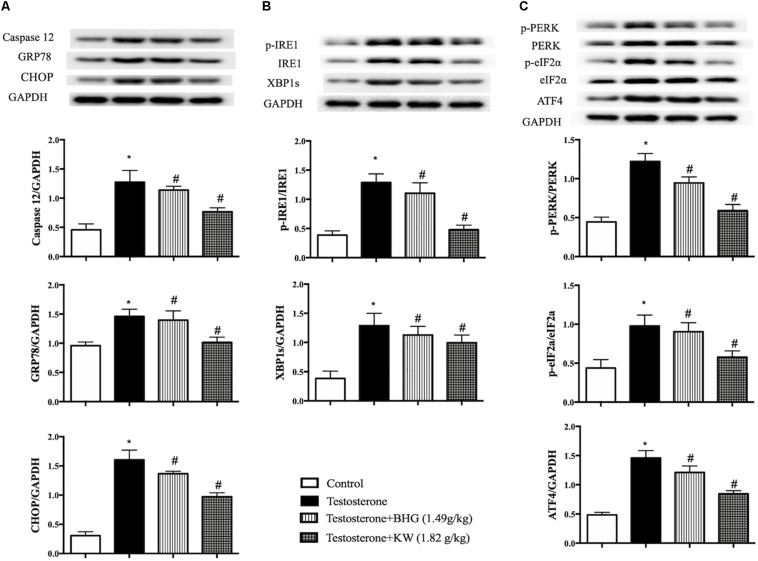
Effect of KW on ER stress in GCs induced by testosterone. **(A)** Presented are the representative western blotting images of caspase-12, GRP78, CHOP with the quantification showing below; **(B)** presented are the representative western blotting images of p-IRE1, IRE1, XBP1s; **(C)** presented are the representative western blotting images of p-PERK, PERK, p-eIF2α, eIF2α, and ATF4 with the quantification showing below. Results are presented as mean ± SEM. *P < 0.05 vs. Control group; ^#^*P* < 0.05 vs. Testosterone group, *n* = 3 for western blot. Testosterone + BHG (1.49 g/kg) denotes the cells treated with serum from animals receiving BHG at 1.49 g/kg, Testosterone + KW (1.82 g/kg) denotes the cells treated with serum from animals receiving KW at 1.82 g/kg.

Caspase-12, GRP78, and CHOP are indicative proteins for ER stress. As shown in Figure 11A, testosterone increased the expression of caspase-12, GRP78, and CHOP, which was decreased by KW, but not by BHG. We next examined ER stress sensors IRE1 and PERK in different group of GCs. Western blot assay showed that the ratios of phospho-IRE1/IRE1, phospho-PERK/PERK, phospho-eIF2α/eIF2α, and the level of ATF4 expression were increased by testosterone stimulation, which were all ameliorated by KW but not BHG (Figures 11B,C), indicating that KW ameliorated the ER stress-dependent apoptotic signaling pathway induced by testosterone whereas, BHG did not show effect on this signaling.

## Discussion

The present study demonstrated that BHG and KW ameliorated DHEA-induced PCOS symptoms such as irregular estrous cycles, polycystic ovary and high levels of testosterone and insulin in serum. Particularly, treatment with BHG and KW both improved the outcome of pregnancy, increased number of pups. These results verified the efficiency of BHG and KW in treatment of PCOS in clinic.

Increasing studies in PCOS patients and DHEA-induced PCOS animal model indicates apoptosis of GCs as one of the main manifestations of PCOS, which is involved in the dysfunction of GCs (Sun et al., 2017; Takahashi et al., 2017; Zhao et al., 2018), and emerges as a target for improvement of PCOS symptoms (Chang et al., 2013; Lim et al., 2017b; Lima et al., 2018). However, the signaling pathways implicated in apoptosis of GCs of PCOS have not been fully elucidated. Consistent with previous reports, the present study revealed a prominently increased apoptosis in GCs in rats in response to DHEA stimulation. Furthermore, signaling protein analysis found that the apoptosis in GCs after DHEA was mediated by mitochondria- and ER stress-dependent pathways, but without involvement of death receptor-dependent pathway. This result implies that mitochondria and ER were impaired in GCs of PCOS, as confirmed by the findings that DHEA caused an increase in cytochrome C release from mitochondria and a dysfunction of mitochondria respiratory chain, as well as an upregulated ER stress protein. The dysfunctional mitochondria and ER stress may also account for the oxidative stress after DHEA observed in the present study, which is in line with the observations of others in patients (Devine et al., 2012; Karuputhula et al., 2013; Meng et al., 2013; Wang et al., 2019). Interestingly, we observed that both BHG and KW may attenuate the apoptosis in GCs after DHEA stimulation, but via different pathway with BHG targeting mitochondria-dependent apoptotic pathway while KW targeting ER stress-dependent apoptotic pathway. The findings in PCOS model was verified by *in vitro* study using GCs. This result shows that although BHG and KW are both effective in treatment of PCOS, but the underling mechanism likely differs from each other. More importantly, this finding provides clue for development of novel management to deal with PCOS.

In physiological condition, testosterone originates approximately equally from ovaries and adrenal grand. In ovaries, DHEA, the intermediate metabolite, transforms to androstenedione in theca cells, which is in part metabolized to testosterone in theca cells while remainder is converted to estrone in GCs (Rosenfield and Ehrmann, 2016). It is predicable that DHEA administration results in an increase in testosterone in plasma, as the case in present study, since the source for synthesis of testosterone has increased. On the other hand, we observed an increased apoptosis of GCs after DHEA, which is likely to contribute in part to the increase in DHEA-caused elevation of testosterone, as the apoptotic GCs protect androstenedione from converting to estrone thus increase the source for synthesis of testosterone. This notion is testified by the fact that BHG and KW attenuated the apoptosis in GCs, meanwhile, decreased the level of testosterone. This result is in accordance with a recent report showing that dysfunction of GCs may contribute to excessive secretion of androgen and abnormal follicles formation (Xu et al., 2011), and further documents the importance of apoptosis of GCs in the development of PCOS. Our *in vitro* experiment showed that testosterone decreased the GCs viability and increased the GCs apoptosis, both BHG and KW are effective in ameliorating the alterations of GCs induced by testosterone via different anti-apoptotic pathway.

The present study revealed that BHG and KW treatment protected insulin form increase in PCOS, this is likely attributable to their ability to decrease the level of testosterone, since hyperandrogen has been reported to induce hyperinsulinemia through the regulation of adiponectin, tumor necrosis factor, interleukin-6, leptin, and other factors (Escobar-Morreale, 2018).

In the present study, we tested three different doses for each medicine with the middle one being the equivalent human dose in clinic. For most parameters examined, no significant dose-dependent effect was observed for either medicine. This result may imply that the two medicines have a larger dose tolerance in term of the endpoints examined. However, it should be very careful to extrapolate this result to clinic, since high dose may exhibit some adverse side effect, as suggested by the fact that BHG at high dose seemed less effective at least for some parameters tested, and the morphology of ovarian tissue exposed to BHG at low and medium dose seemed to be better than that to BHG at high dose. Moreover, both drugs contain dozens of components. Another limitation of the present work is that no study is performed to explore the effective compound (s) responsible for the effect observed, although some compositions of both drugs were detected in the plasma after administration (Supplementary Material 3).

In summary, the present study demonstrated the efficiency of BHG and KW in treatment of PCOS in rat induced by DHEA, this effect is likely associated with attenuation of GCs apoptosis protecting testosterone from increase. BHG and KW exerted protective effect on GCs apoptosis via different pathway with BHG interfering in mitochondria-dependent apoptotic pathway while KW targeting ER stress-dependent apoptotic pathway.

## Perspective

Polycystic ovary syndrome accounts for about 70% of anovulatory infertility cases. BHG and KW are currently used in China to cope with PCOS with efficiency, however, the underlying mechanisms remain unknown. The present study confirmed the effect of the two medicines in treatment of rat PCOS model induced by DHEA and found that both medicines exhibit potential to attenuate GC apoptosis but via different pathway with BHG targeting mitochondria-dependent apoptotic pathway while KW targeting ER stress-dependent apoptotic pathway. This potential may contribute to downregulation of testosterone thus relieve the PCOS. These results provide scientific basis for the use of the two medicines in practice and promote their application. The finding that GC apoptosis in PCOS implicated mitochondria- and ER-stress dependent apoptotic pathways without involvement of death receptor-dependent apoptotic pathway offered clue for searching novel option to fight against PCOS.

## Data Availability Statement

All datasets generated for this study are included in the article/Supplementary Material.

## Ethics Statement

The animal study was reviewed and approved by the Committee on the Ethics of Animal Experiments of Peking University Health Science Center.

## Author Contributions

YX and C-SP performed the experiments, analyzed the data, and wrote the manuscript. QL provided scientific guidance and oversight. H-LZ contributed to the animal experiments. LY contributed to confocal imaging. GA participated in western blot assay of GCs experiments. X-YW and L-LY participated in the preparation of sample for LC-MS analysis. J-YF and J-YH revised the manuscript. DL and J-YH supervised the research and provided key research directions. All authors read and agreed with the final article.

## Conflict of Interest

The authors declare that the research was conducted in the absence of any commercial or financial relationships that could be construed as a potential conflict of interest.
