# Supplementary material for: The Ameliorating Effects of Bushen Huatan Granules and Kunling Wan on Polycystic Ovary Syndrome Induced by Dehydroepiandrosterone in Rats
Source: Front Physiol. 2021 Mar 8;12:525145. doi: 10.3389/fphys.2021.525145 (PMC7982847; doi:10.3389/fphys.2021.525145)
Supplement: Supplementary file 1 [file Data_Sheet_1.docx]

**Supplementary materials**

**S1：Table 1. Composition of BHG**

| Species | Chinese name | Part used | Family | Weight percent |  |
| --- | --- | --- | --- | --- | --- |
| Epimedium brevicornu Maxim | Yinyanghuo | Herba | Berberidaceae | 5.9 |  |
| Cuscuta chinensis Lam | Tusizi | Seed | Convolvulaceae | 8.2 |  |
| Atractylodes lancea | Cangzhu | Root | Asteraceae | 17.2 |  |
| Pinellia ternata | Banxia | Rhizome | Araceae | 13.0 |  |
| Citrus reticulata Blanco | Chenpi | Pericarp | Rutaceae | 13.0 |  |
| Cyperus rotundus L | Xiangfu | Rhizome | Cyperaceae | 3.9 |  |
| Salvia miltiorrhiza Bge | Danshen | Root | Labiatae | 14.1 |  |
| Cornu cervi degelatinatum | Lujiaoshuang | Antler bone residue | Cervidae | 3.9 | |
| Arisaema cum bile | Dannanxing | Rhizome, bile | Araceae | 7.8 | |
| Coptis chinensis Franch | Huanglian | Rhizome | Ranunculaceae | 13.0 | |

| Species | Chinese name | Part used | Family | Weight percent |
| --- | --- | --- | --- | --- |
| Cyperus rotundus L | Xiangfu | Rhizome | Cyperaceae | 15.7 |
| Radix glycyrrhizae | Gancao | Rhizome | Papilionaceae | 1.5 |
| Radix cynanchi atrati | Baiwei | Root | Asclepiadaceae | 3.0 |
| Herba leonuri | Yimucao | Acrial part | Lamiaceae | 3.0 |
| Radix scutellariae | Huangqi | Root | Leguminosae | 3.0 |
| Celosia cristata | Jiguanhua | Inflorescence | Amaranthaceae | 3.0 |
| Radix ophiopogonis | Maidong | Earthnut | Liliaceae | 3.0 |
| Fructus Schisandrae chinensis | Wuweizi | Fruit | Magnoliaceae | 3.0 |
| Radix rehmanniae | Dihuang | Earthnut | Scrophulariaceae | 3.0 |
| Carthamus tinctorius L | Honghua | Flower | Asteraceae | 3.0 |
| Akebia quinata | Mutong | Caculis | Lardizabalaceae | 2.1 |
| Atractylodes macrocephala | Baizhu | Rhizome | Asteraceae | 3.0 |
| Halloysitum rubrum | Chishizhi | Kaolinite | Silicate | 3.0 |
| Poria cocos | Fuling | Sclerotium | Polyporaceae | 3.0 |
| Magnolia officinalis | Houpu | Dried bark, root bark, twig bark | Magnoliaceae | 2.1 |
| Cistanche salsa | Roucongrong | Fleshy stem | Orobanchaceae | 3.0 |
| Cynanchum otophyllum | Baishao | Root | Paeoniaceae | 3.0 |
| Nepeta cataria L | Jingjie | Stem leaf, spica | Labiatae | 2.1 |
| Cortex moutan | Mudanpi | Root bark | Ranunculaceae | 3.0 |
| Colla Corii Asini | Ejiao | Donkey skin | Equidae | 3.0 |
| Angelica sinensis | Danggui | Root | Lamiaceae | 3.0 |
| Ligusticum sinense Oliv | Gaoben | Rhizome, root | Umbelliferae | 2.1 |
| Panax ginseng C.A.Mey | Hongshen | Root | Araliaceae | 3.0 |
| Deerhorn Glue | Lujiaojiao | Antler | Cervidae | 3.0 |
| Fritillaria cirrhosa D. Don | Chuanbeimu | Stem | Liliaceae | 3.0 |
| Nacre Concha Margaritifera Usta | Moyao | Resin | Burseraceae | 3.0 |
| Amomum villosum Lour | Sharen | Fruit | Zingiberaceae | 3.0 |
| Corydalis | Yanhusuo | Tuber | Papaveraceae | 3.0 |
| Foeniculum vulgare | Xiaohuixiang | Fruit | Umbelliferae | 3.0 |
| Colla Carapacis et Plastri Testudinis | Guijiajiao | Carapace | Testudinidae | 3.0 |
| Ligusticum wallichii | Chuanxiong | Rhizome | Apiaceae Lindl | 3.0 |
|  |  |  |  |  |
|  |  |  |  |  |

**S2: Table 2. Composition of KW**

**S3: The main effective ingredients of BHG and KW**

**1. Sample preparation**

Plasma sample of 100μL was taken from each animal, 3 rats in each group at each time point, added with 600μL of acetonitrile, mixed and centrifuged at 12000rpm for 10min. A volume of 600μL of supernatant was taken and blowed dry, added with 100μL of 5% acetonitrile water to redissolve. The solution was then subjected to LC-MS analysis.

**2. Results**

**2.1 Main effective ingredients of BHG**

The differential peak of blank and post-dose plasma samples is depicted in Figure 1. Among the reported compounds in each prescription, one compound was found in dosed plasma (Figure 2). The mass information of the absorbed components in rat plasma is shown in Table 1.

Figure 1 TIC of rat plasma in positive mode (oral administration of BHG**)**

1
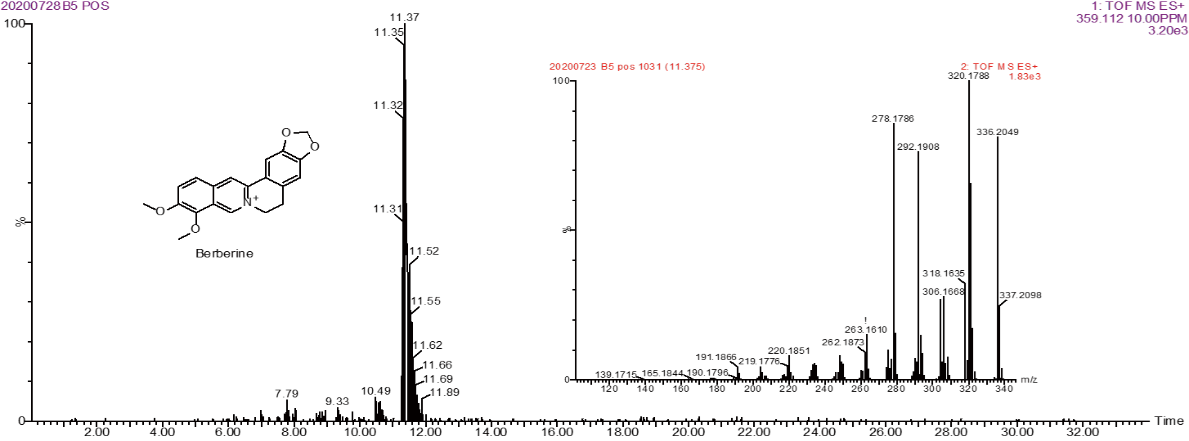


Figure 2 Extracted ion chromatogram and MS/MS fragmentations of Berberine

**Table 3** The MS information of absorbed component of BHG in rat plasma

| Retention Time | m/z | MS/MS | Molecular Formula | | Compound | | Source |
| --- | --- | --- | --- | --- | --- | --- | --- |
| 11.37 | 359.1123[M+Na]^+^ | 320.1788、292.1908、278.1786 | | C_20_H_18_NO_4_ | | Berberine | *Coptis chinensis* Franch. |

**2.2 Main effective ingredients of KW**

The differential peak of blank and post-dose plasma samples of KW is presented in Figure 3. Among the reported compounds in the prescription, 2 compounds were found in dosed plasma. (Figure 4 and Figure 5). The mass information of the absorbed components in rat plasma is shown in Table 2.

Figure 3. TIC of rat plasma in positive mode (oral administration of KW)

Figure 4. Extracted ion chromatogram and MS/MS fragmentations of Paeonoside

Figure 5. Extracted ion chromatogram and MS/MS fragmentations of Tetrahydropalmatine

**Table 4.** The MS information of absorbed components in rat plasma

| Retention Time | m/z | MS/MS | Molecular Formula | Compound | Source |
| --- | --- | --- | --- | --- | --- |
| 6.22 | 351.1049 [M+Na]^+^ | 351.1049 167.0706 | C_15_H_20_O_8_ | Paeonoside | *Paeonia suffruticosa* Andr. |
| 7.22 | 356.1833 [M+H]^+^ | 356.1833  192.1049 | C_21_H_25_NO_4_ | Tetrahydropalmatine | *Corydalis yanhusuo* W.T.Wang |

**S4: The dose safety of drugs**

**Results:** Compared with control, different concentrations of BHG and kW had no significant effect on serum ALT (A), AST (B), Cr (C) and BUN (D), suggesting that they had no toxicity on liver function and renal function**.**

A B


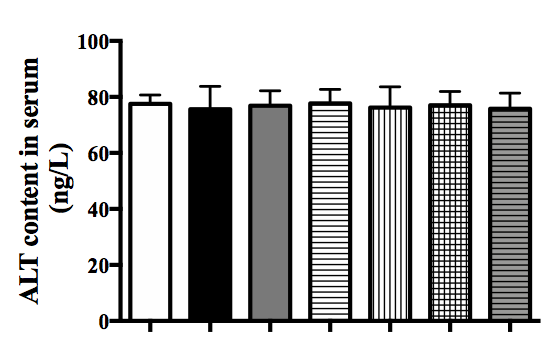

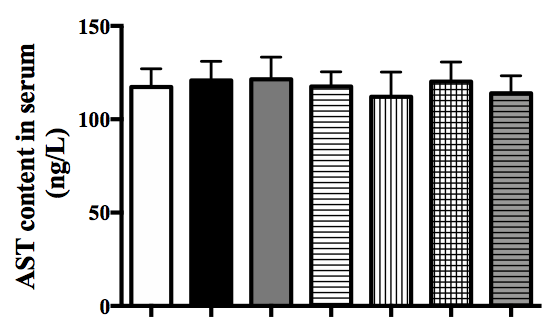


C D


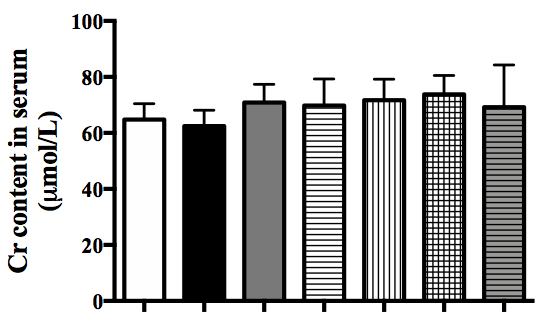

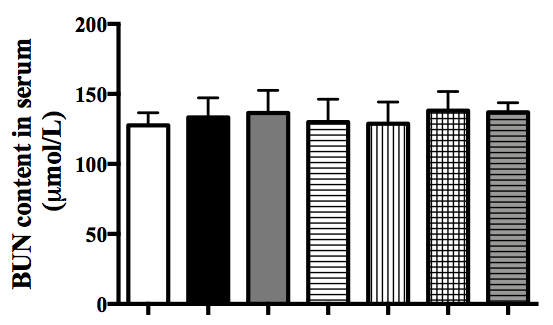


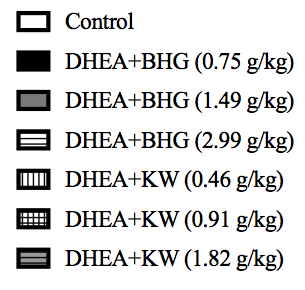


Figure 6: The effects of different concentrations of BHG and kW on serum ALT (A), AST (B), Cr (C) and BUN (D). Results are presented as mean ± SEM. n = 7.
